# The Role of Pentraxin 3 in Gastrointestinal Cancers

**DOI:** 10.3390/cancers15245832

**Published:** 2023-12-14

**Authors:** Monika Zajkowska, Barbara Mroczko

**Affiliations:** 1 Department of Neurodegeneration Diagnostics, Medical University of Bialystok, 15-269 Bialystok, Poland; mroczko@umb.edu.pl; 2 Department of Biochemical Diagnostics, Medical University of Bialystok, 15-269 Bialystok, Poland

**Keywords:** PTX-3, colorectal, small intestine, stomach, pancreatic, liver, gallbladder, esophageal, GI cancers

## Abstract

**Simple Summary:**

Gastrointestinal (GI) cancers are all malignant lesions affecting the esophagus, stomach, liver and intrahepatic bile ducts, gallbladder, pancreas, and small and large intestines. With the rising number of new GI cancer cases, we set out to clarify the role of Pentraxin-3 in their development. This was due to the increasing need to investigate novel biomarkers and therapeutic targets for the identification and treatment of cancerous lesions. Throughout the course of several gastrointestinal malignancies, significant changes in the concentration, tissue, and gene expression of this parameter were observed. In conclusion, the tested protein may be very helpful in the early identification of gastrointestinal malignancies and useful as a therapeutic target.

**Abstract:**

Gastrointestinal cancers have become a huge problem worldwide as the number of new cases continues to increase. Due to the growing need to explore new biomarkers and therapeutic targets for the detection and treatment of cancerous lesions, we sought to elucidate the role of Pentraxin-3 in the progression of cancerous lesions, as it is involved in the process of angiogenesis and inflammation. Statistically significant changes in the concentration of this parameter have emerged in many gastrointestinal cancer patients. Moreover, it is related to the advancement of cancer, as well as processes leading to the development of those changes. In the case of studies concerning tissue material, both increased and decreased tissue expression of the tested parameter were observed and were dependent on the type of cancer. In the case of cell lines, both human and animal, a significant increase in Pentraxin 3 gene expression was observed, which confirmed the changes observed at the protein level. In conclusion, it can be assumed that PTX3, both at the level of gene expression and protein concentrations, is highly useful in the detection of gastrointestinal cancers, and its use as a biomarker and/or therapeutic target may be useful in the future.

## 1. Gastrointestinal Cancers

Gastrointestinal tract (GI) cancers are all malignant changes affecting such organs as the esophagus, stomach, liver and intrahepatic bile ducts, gallbladder, pancreas, and small intestine; all changes in sections of the large intestine (colon and rectum) are collectively known as colorectal cancer and anus cancer [1,2]. Those cancers are the most common among men and the second most common (after breast cancer) in women. The most common type of gastrointestinal cancer is colorectal cancer, and the most life-threatening is pancreatic cancer, as only 15–20% of patients suffering from this type of malignancy are eligible for surgical treatment, which gives a chance for permanent recovery [2].

According to the International Agency for Research on Cancer (IARC) data presented for 2020, nearly 5.2 million new cases and over 3.6 million deaths concerning all gastrointestinal cancers have been estimated around the world for both sexes [3,4,5]. Cancer remains a serious worldwide problem, as it is the second leading cause of death after cardiovascular diseases. Carcinogenesis is a well-known multifactorial process involving both genetic and environmental perturbations [2]. Genetic factors influencing the development of neoplastic changes in the gastrointestinal tract, especially in patients under the age of 50, mainly include overlapping changes in the DNA. The literature describes many cases of diseases in the family related to genetic predisposition. Moreover, it is estimated that about 20% of GI cancers may be of genetic origin. An example of such a disease is Lynch syndrome (colorectal cancer not associated with polyposis), familial adenomatous polyposis, or MUTYH-associated polyposis [6,7]. The most important environmental factors include microorganisms (H. pylori, hepatitis viruses), obesity and an incorrect diet (especially high-fat and low in plant products, vitamins, and minerals), smoking, stress, alcohol abuse, or low physical activity [8].

Esophageal cancer (EC) is a malignancy that occurs in the first sections of the digestive system. The most common histological type of esophageal cancer is squamous cell carcinoma, which is responsible for about 90% of deaths in Asian countries [9]. It occurs mainly in the upper and middle parts of the organ. The glandular type is most often located in the lower part of the esophagus. The main factors that increase the risk of this cancer, especially the squamous cell type, are, in most cases, smoking and alcohol consumption, which most likely change the genome in genetic and epigenetic terms [10]. Other factors include previous treatment for squamous cell carcinoma of the head and neck or lung, esophageal burn, or Plummer–Vinson syndrome. The risk of developing adenocarcinoma most often manifesting in the lower part of the esophagus increases in Barrett’s esophagus, gastroesophageal reflux disease, obesity, and smoking. Interestingly, in the USA, there was a significant increase in the incidence of this type of cancer (over six times) [9,10,11]. In 2020 alone, over 600,000 new cases and almost 550,000 deaths due to this cancer were recorded worldwide. Based on the bottom epidemiological data provided by GLOBOCAN, it can be observed that most of the mentioned cases concern men (about 2.2 times more cases), and the main endemic area of this disease is the Asian continent [3,4].

Gastric cancer (GC) arises from the epithelial tissue of the mucosa that lines the inside of the stomach. It is the fifth cancer in the world in terms of incidence and third in terms of mortality. This disease mainly affects men—compared with women, they are affected about twice as often. In 2020, almost 1.1 million new cases were detected and over 760,000 deaths due to this cancer were recorded worldwide, from which approximately 75% concerned the Asian continent [3,4,12]. The diagnosis of gastric cancer is usually made after the age of 50, although in some cases, the disease may develop earlier. There are several histological subtypes of malignant gastric neoplasms, of which the most common (up to 95% of cases) is adenocarcinoma. Further classification of the disease depends on the morphological structure of the neoplastic tissues. The reasons for the development of gastric cancer are quite diverse and include many environmental, behavioral, and genetic factors [12]. However, it is estimated that the main factors are related to, among others, health habits and diet. Cigarette smoking leads to a decrease in the level of vitamin C, which counteracts the process of carcinogenesis, while the substances contained in tobacco smoke, by directly irritating the gastric musculature and submucosal nerve plexuses, cause the failure of the mechanisms controlling peristalsis. This may result in the regurgitation of duodenal contents into the stomach, inflammation of the mucous membrane, and its biliary inflammation, which may affect the formation of neoplastic changes in glandular cells, starting from the inflammatory atrophy of the mucous membrane, through metaplasia, dysplasia, and malignant transformation [13,14]. Infections deserve special attention. Helicobacter pylori or Epstein–Barr infections are risk factors for stomach cancer [13,15]. The most common malignant tumor of the stomach is adenocarcinoma. Lymphomas, gastrointestinal stromal tumors (GISTs), sarcomas, and neuroendocrine tumors (NETs) are much less common [13].

Hepatocellular carcinoma (HCC) is the most common, accounting for approximately 90% of cases of primary liver tumors. The remaining 10% relate to tumors originating from the intra- and extrahepatic bile ducts. The incidence of this type of cancer varies in different regions of the world, but the highest incidence, as in the case of most gastrointestinal cancers, is in the Far East, which is associated with the high incidence of hepatitis B (endemic region) [16]. In European countries and the USA, the incidence of HCC is mainly associated with the prevalence of hepatitis C. The disease is most often diagnosed in patients over 60 years of age and is more than twice as common in men. Additional risk factors include alcohol consumption, metabolic diseases, such as obesity and diabetes, and aflatoxin exposure. Some diseases can also lead to the development of HCC, including cirrhosis, non-alcoholic steatohepatitis (NASH) connected with metabolic syndrome or diabetes mellitus, as well as non-alcoholic fatty liver disease (NAFLD) [17,18]. In 2020, more than 900,000 new cases and approximately 830,000 deaths from liver cancer were detected, with more than 70% of cases occurring in the Asian continent [3,4,5,17].

Gallbladder cancer (GBC) is a malignant tumor originating from the epithelial cells of the gallbladder mucosa. When GBC is diagnosed at advanced stages of its development, it is characterized by a very poor prognosis, which is connected to its aggressive behavior and limited therapeutic options [19,20]. This tumor coexists in up to 90% of patients with cholelithiasis. It is believed that chronic cholecystitis leads to the initiation of neoplastic processes. In 2020, there were over 115,000 cases and almost 85,000 deaths due to gallbladder cancer. Interestingly, in the case of this cancer, the incidence and death rates are about twice as high in women. About 70% of cases were detected in Asia, while in Europe, less than 11% of all cases were detected [3,4,5]. Risk factors for GBC include, among others, diabetes, high BMI, the consumption of large amounts of sweetened beverages, toxins, chronic bacterial infections, gallstones, and polyps [20,21,22].

Pancreatic cancer (PC) does not cause any symptoms for a long time. Some of the first symptoms are general weakness, lack of appetite, early satiety, upper abdominal discomfort, and bloating. As the disease progresses, other symptoms appear. These include jaundice, progressive weight loss, and abdominal pain radiating from the belly button to the spine. It is one of the most frequently fatal cancers. The basis of diagnosis is imaging tests, especially computed tomography [23,24]. It is one of the most lethal cancers in both men and women. The main risk factors for its development include: smoking, obesity, high consumption of saturated fat (of animal origin), chronic pancreatitis (often associated with alcohol abuse), diabetes, as well as advanced age, blood group (especially type B), family history, and genetic susceptibility [25,26,27,28]. Only in 2020 were almost 500,000 new cases and slightly fewer (over 466,000) deaths detected, which confirms the high mortality rate associated with the detection of pancreatic cancer. It affects a comparable number of women and men, most often inhabiting Asia (about 47–48%), Europe (28%), and North America (11–12%) [3,4,25].

Small bowel cancer is considered as a rare tumor and accounts for about 3% of all GI cancers. There are several risk factors that can contribute to the development of small bowel cancer. The first is age—the disease is most often diagnosed in patients between 60 and 65 years of age, depending on the histological type. Smoking cigarettes and drinking a lot of alcohol are also important. Furthermore, small intestine cancer is often associated with Crohn’s disease, celiac disease, polyps, and gastrointestinal polyposis syndrome. In addition, the better-known risk factors include inherited Peutz–Jeghers genetic syndrome, HNPCC (hereditary colorectal cancer without polyposis), or FAP (familial adenomatous polyposis) [29,30,31].

Colorectal cancer (CRC) is the largest group of GI cancers. In 2020, this type of cancer affected nearly 1.2 million people around the world (colon and rectum; about 1.15 million and more than 730,000 cases, respectively), and more than 900,000 died from this cancer (colon and rectum, about 580,000 and more than 330,000 cases, respectively) [3,4,32]. This ranks this cancer as the third most morbid and the second most fatal among all cancers. Men are affected more often. The symptoms of colorectal cancer vary depending on the stage and location of the tumor. In right-side bowel cancer, the symptom is usually occult bleeding with progressive anemia. When the cancer is located on the left side, the most common symptom is overt bleeding from the lower gastrointestinal tract and a change in bowel habits [33,34]. Genetic factors are the main cause of approximately 15–30% of all cases of the disease. These include Lynch syndrome, a previous history of colorectal cancer or excision of polyps with a high degree of dysplasia, a family history of colorectal cancer, familial adenomatous polyposis syndrome, or inflammatory bowel diseases. Environmental risk factors for the development of colorectal cancer are associated with the majority of cases. These include age, obesity, smoking, low physical activity, and a diet low in fruit and vegetables, as well as calcium, fiber, and selenium, and rich in animal fats and high in calories [32,33,34].

Cancer of the last part of the gastrointestinal tract is called anal tumor. It is estimated that it accounts for less than 3% of all GI malignancies. The peak incidence of anal cancer falls after the age of 60, and it more often affects women. The main histological subtype is squamous cell carcinoma of the anus, caused mostly by oncogenic HPV types. HPV has been associated with more than 90% of all cases of this type of cancer. The most common symptom of anal cancer is rectal bleeding (fresh blood). Other clinical symptoms of anal cancer include: anal pruritus, pain during defecation, tenesmus, the feeling of a foreign body in the anus, gas incontinence, palpable lump, and enlarged inguinal lymph nodes. In 2020, around 50,000 cases and fewer than 20,000 deaths from this cancer were diagnosed worldwide [3,4,35,36].

## 2. Pentraxin Family

Pentraxins are a family of proteins with a similar configuration. They have the same domain and monomers in their structure, which are arranged discoidally in the form of a pentamer, hence the name of the described proteins. Family members are characterized by, located at the C-terminus, an amino acid sequence exceeding 200, called the pentraxin domain. Based on the length of the protein sequence, the pentraxin family can be divided into two subfamilies: short (classical) and long (fusion) pentraxins. The short pentraxin subfamily includes C-reactive protein (CRP) and serum amyloid P component (SAP), while the long pentraxin subfamily includes neuronal pentraxins and their receptor (NPTX1, NPTX2, and NPTXR), as well as pentraxin 3 (PTX3) and pentraxin 4 (PTX4). The long pentraxins are approximately twice as large as the short pentraxins and have an unrelated long N-terminal sequence. This variation in the structure of family members may explain the difference in their functions [37,38]. Research conducted to date has revealed the functions of specific members of the pentraxin family. Members such as CRP and SAP have previously been reported as mediators in the regulation of the human immune system. Their functions include preventing pathogen invasion, eliminating mutated cells, and inducing inflammatory processes. Neuronal pentraxins are mainly involved in the progress of the central nervous system and neurodegenerative diseases. Interestingly, PTX3 not only participates in the activation of the immune system, but has also been connected to cancer progression [37].

The first mentions of proteins belonging to the pentraxin family concerned the C-reactive protein (CRP) and appeared in the 1930s. However, more reports on pentraxins were published in the early 1980s, when the first references of other members of this family appeared. Short pentraxins consist of protomers with a mass of about 25 kDa, while in the case of long pentraxins, these components have a mass of about 42 kDa, and their sequence coincides with the sequence of CRP in about 25–30% [38]. C-reactive protein was firstly identified as a protein that precipitated C-polysaccharide isolated from the *S. pneumoniae* cell wall in the presence of calcium ions (Ca^2+^). In humans, it is considered as an acute phase protein, as its concentrations can increase even 1000-fold in the course of severe inflammation. As is well known, elevated CRP levels are a useful indicator of the development of selected human diseases, including bacterial and fungal infections, cardiovascular diseases (hsCRP), autoimmune diseases, and cancer [39]. Currently, many medical decisions regarding the inclusion of antibiotic therapy are made based on the CRP concentration, as it can determine whether we are dealing with a viral or bacterial infection, which, in the case of small children, may be crucial [40]. Interestingly, there are also works describing the changes in the concentrations of this protein during exposure to long-term stress and during COVID-19 [41,42]. It has been proven that increasing CRP concentrations are associated with the development of this disease and can serve as an early predictor of severe COVID-19 [43].

Serum amyloid P component (SAP), also called pentraxin 2 (PTX2), was first identified in pathological deposits in vivo as the serum form of AP (amyloid P component). SAP shows similarity in structure to CRP in about 66%. Similar to CRP, the SAP gene is located on chromosome 1 and is secreted by liver cells. So far, it has been found in all types of amyloid deposits that disrupt tissue architecture. It is therefore believed that SAP plays an important role in the pathogenesis of a related group of diseases, called amyloidosis. Interestingly, SAP is elevated in the brains of Alzheimer’s disease (AD) patients and is associated with amyloid plaques, as it is related to wound healing. In the case of Parkinson’s disease (PD), SAP is identified as part of a potential diagnostic pattern. However, the largest number of reports on SAP concerns cardiovascular diseases [44,45].

Pentraxin 3 (PTX3) is a multi-functional parameter with multifaceted regulatory functions in inflammatory processes involved in the organization of ECM (extracellular matrix) and remodeling [46]. A rise in blood PTX3 levels acts as a marker for the onset of inflammation. The fact that the maximum PTX3 level elevates slightly earlier than the CRP level reveals that PTX3 is a sensitive inflammation-related marker [37]. Furthermore, it has been shown that PTX3 may play a role as an extrinsic tumor-suppressor gene by controlling complement-dependent and macrophage-associated tumor-promoting inflammation [47]. Its expression has been found, among others, in lung cancer, glioma, ovarian cancer, myxoid liposarcoma, prostate carcinoma, esophageal squamous cell carcinoma, and pancreatic cancer [48]. Interestingly, PTX3 has been also shown as a biomarker (prognostic indicator of short-term mortality) of clinical PASC (post-acute sequelae of SARS-CoV-2), also known as post-COVID syndrome [49,50]. Some other authors revealed the involvement of PTX3 in the tumor microenvironment in promoting cancer metastasis, invasion, and stemness. Those findings not only described the biological significance of PTX3 in tumorigenesis, but also suggested that PTX3 may be a key pro-tumorigenic player in the tumor microenvironment [51]. It has also been proven that PTX3 can act as an FGF2 antagonist, as it has the possibility to bind to FGF2 with high affinity and specificity. This interaction prevents the binding of FGF2 to its cognate tyrosine kinase receptors, leading to the inhibition of the angiogenic activity of the growth factor [52].

Pentraxin 4 (PTX4) was identified recently in 2010; therefore, there are few studies describing the action of this protein [53]. Its high tissue expression under physiological conditions has been established in bone marrow, the small intestine, and testes. Interestingly, PTX4 is not induced by IL-1 or LPS and does not act as an acute-phase protein. Its high expression has been found in traumatic brain injury (TBI) [54,55].

Neuronal pentraxins are mainly expressed in neurons (NPTX1), brain tissues, the testicles, pancreas, and muscles (NPTX2). Neuronal pentraxin receptor (NPTXR) is mostly expressed in the brain and binds to taipoxin, TCBP49, NPTX1, and NPTX2. It activates various downstream signal transduction processes [37]. Some mental illnesses, including bipolar disorder, central precocious puberty, anxiety, depression, childhood-onset mood disorders, and schizophrenia, have been linked to abnormal expression of the neuronal pentraxins [37,56,57]. Neurodegenerative diseases, including Alzheimer’s disease (AD) and Parkinson’s disease (PD), are also linked to neuronal pentraxins. Recently, researchers reported that the NPTXR protein in cerebrospinal fluid may play a role as a potential biomarker of AD progression and may be useful in determining the efficiency of treatment in clinical trials. NPTX1 may also play a crucial role in the pathophysiology of PD [58,59,60,61].

## 3. Role of Pentraxin-3 in GI Cancers

**Figure 1:**
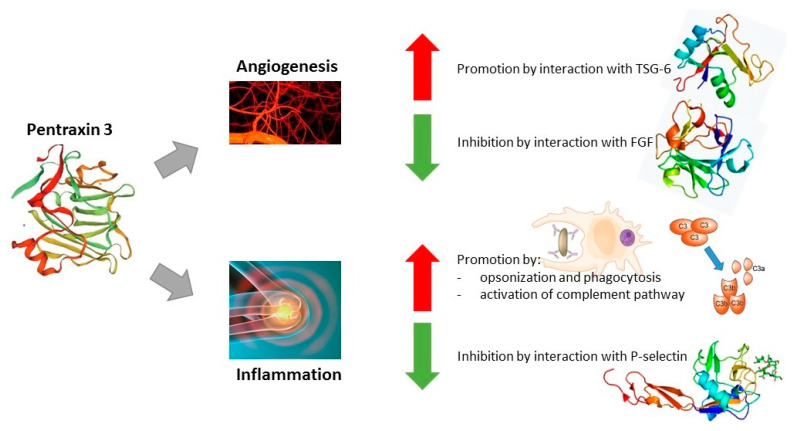
Functions of Pentraxin 3. Abbreviations: ↑—promotory effect of PTX3; ↓—inhibitory effect of PTX3.

Although Pentraxin 3 shares sequence similarity with CRP and SAP genes, there are considerable differences in the cell types that are responsible for their synthesis, as well as stimulation for its production. PTX3 is expressed and released by different cell types at the site of inflammation, e.g., innate immune cells, stromal cells, as well as cancer cells, in contrast with CRP or SAP, which are primarily produced within the liver due to IL-6 mediated inflammation. Multiple proinflammatory mediators may trigger PTX3 expression, e.g., IL-1β and TNFα, toll-like receptor agonists, HDL, IL-10, and various microbial components (e.g., LPS). As a result, PTX3 can be released from neutrophil secondary granules, reaching the highest concentration 4 to 6 h after stimulation [47,62]. Pentraxin 3 PTX3 also plays a role in tissue regeneration and remodeling during angiogenesis by interacting with P-selectin, modulating complement activation, and remodeling the provisional fibrin-rich extracellular matrix. Its N-terminal domain has high-affinity binding ability for FGF (fibroblast growth factor). By complex formation with FGF, PTX3 efficiently prevents the formation of the HSPG/FGF/FGFR ternary complex, causing a blockage of FGF-dependent endothelial cell proliferation in vitro and blood vessel growth in vivo. This mechanism effectively reduces inflammation and cancer-associated angiogenic activity [62,63]. It has been shown that PTX3 stimulates cancer cell proliferation, apoptosis, and metastasis in a variety of malignancies by acting through the PI3K/AKT/mTOR signaling pathway. Surprisingly, PTX3 reduces the production of cell cycle-related proteins during the G2/M phase, which, in turn, prevents cancer from proliferating and metastasizing. Additionally, in steroid hormone-regulated cancers, PTX3 can bind to the FGF8b receptor and prevent the proliferation of cancer cells [37,62]. Therefore, it can be considered that this protein has both pro- and anti-cancer effects. The functions of PTX3 have been summarized in Figure 1. 

As a result of an in-depth analysis of the available literature regarding the usefulness and role of Pentraxin 3 in gastrointestinal cancers, we managed to determine that the evaluation of this protein concentration in the blood (serum or plasma) of patients with colorectal cancer is extremely useful. Research conducted by Liu et al. [64] proved that the concentration of this parameter differed significantly between the study group consisting of 263 patients with colorectal cancer and the control group (126 healthy volunteers). Moreover, the concentration of this parameter increased significantly with the stage of advancement of the examined cancer. After statistical analysis, the authors determined the optimal cut-off point for PTX3 to be at the level of 12.6 ng/mL (sensitivity 68%; specificity 71.7%) using the ROC (receiver operating characteristic) curve with an AUC of 0.732. Interestingly, based on the afore-mentioned cut-off point, it was determined that patients with higher PTX3 concentrations had a poorer five-year overall survival rate compared with patients with lower concentrations of this protein. Owing to the conducted analyses, it was possible to establish that PTX3 could be evaluated for an optimal risk stratification and was an independent predictor for the postoperative survival of CRC patients [64]. Similar conclusions were reached by Zhang et al. [65]. This research was conducted on 184 patients with diagnosed CRC and a control group consisting of 216 healthy participants and confirmed that Pentraxin 3 occurs in higher concentrations in the blood of cancer patients [65]. Importantly, the authors repeated the determination of the examined parameter after resection and at the time of relapse. It turned out that, after the procedure, the level of the tested protein decreased significantly, almost to the concentrations observed in healthy volunteers (perhaps this was due to the too-short time from the resection to blood collection), while after observing remission, it increased significantly to the concentrations observed initially. The authors noticed that the tumor-free survival rate for patients from the study group with concentrations higher than 12 ng/mL was significantly lower, which confirmed the reports of Liu et al. [64]. Additionally, the authors observed a positive correlation between the PTX3 concentration and complement components (C3, C4, and 5b9), suggesting that PTX3 levels may reflect immune condition and complement activation in CRC patients [65]. The work of Di Caro et al. [66] regarding the search for prognostic biomarkers of CRC also concerned PTX3 concentrations. The authors conducted research using 69 samples from oncological patients undergoing surgical resection. They showed that PTX3 was the best predictor of prognosis for all parameters tested (better than Li-1, IL-6, IL-10, TNFalpha, CCL2, CXCL8, and VEGF). In summary, the authors noted that their reports suggested the introduction of circulating mediators of inflammation for the detection of CRC progression after surgery and could effectively identify those individuals who have high risk of recurrence, independently of conventional predictor risks; additionally, they can serve as an additional test when making decisions during postsurgical screening [66]. In the work of Papil et al. [67], the researchers also confirmed that, in the case of ongoing cancer (CRC), the concentration of PTX3 is significantly increased. Interestingly, in this study, the authors showed that the concentrations of Pentraxin 3 were significantly correlated with the concentrations of NF-kB and IL-6, which confirmed the involvement of PTX3 in cancer-associated inflammation. In the work of Rubino et al. [68], the authors showed that the expression of PTX3 was under the control of two enhancer genes and that their hypermethylation was responsible for the silencing of PTX3 in CRC. Thus, these genetic elements emerged as important players in the fine regulation of PTX3 expression in physiological and pathological conditions. Similar studies on the silencing of PTX3 were conducted by Bonavita et al. [47]. Researchers have proven that PTX3 acts as an extrinsic oncosuppressor gene both in mouse and human models (cancer cell lines), and the complement system is a key component of tumor-promoting cancer-related inflammation. Contrary to the previously described research, in the work of An et al. [69], it was shown that the concentration of PTX3 in tissue homogenates from CRC patients did not differ significantly between tumor tissue and healthy tissue; however, as the authors themselves admitted when describing the limitations of the study, the freeze–thaw process could have influenced the concentrations of the described proteins. Additionally, as this is the only study describing the concentration of this parameter in cancerous and healthy tissues, it is not certain whether the described results would not be confirmed in subsequent studies, as it is possible that PTX3 does not accumulate in cancerous tissues and its concentration increases only in the blood. While reviewing the literature on CRC, we encountered two cases that used PTX3 as a specific biomarker for the described lesions. In the work of Chen et al. [70], the researchers proved that Orf Virus strain NA1/11 could inhibit CRC growth and metastasis by inducing the apoptosis of CRC cells. This was confirmed by the observation of an increased PTX3 concentration due to virus activity. Interestingly, the work of Mathieu et al. [71] described the usefulness of using alkaloids isolated from the *Amaryllidaceae* plant as anticancer agents. They revealed that those alkaloids decreed cell proliferation and PTX3 concentration (as well as VEGF and MMPs), regardless of TP53 status, which suggests the possibility of their use as multifaceted drug candidates.

In the case of primary liver cancer, we found studies that described the usefulness of PTX3 as a biomarker of HCC in chronic hepatitis B and C virus infections. The work of Deng et al. [72], in which the described research was carried out on a group of over 500 participants, showed that the concentrations of Pentraxin 3 in patients with HCC were higher compared with patients with chronic hepatitis or cirrhosis. Additionally, the concentrations of this protein were higher in the group of patients with HBV compared with healthy patients. The researchers in this study indicated that PTX3 was an independent risk factor of HCC and helped to distinguish HCC from chronic HBV infection, not only in advanced stages, but also in the initial stages, as well as in those patients whose AFP (alpha fetoprotein) levels were negative. The combined analysis of AFP and PTX3 concentrations performed by the authors demonstrated the high diagnostic usefulness of distinguishing HCC from chronic HBV infection (AUC = 0.948). Referring to HCV infection, we found two papers in which the authors described the usefulness of Pentraxin 3. In the paper by Mehanna et al. [73], the authors proved that PTX3 concentrations were the lowest in healthy people, significantly higher in patients with cirrhosis caused by HCV infection, and the highest in patients with HCC induced by HCV infection. This suggests the high usefulness of measuring this parameter and allows for quick intervention at a time frame allowing for the radical treatment of HCC. Moreover, in the work of Carmo et al. [74], the authors proved that there was a significant association between PTX3 polymorphisms and the occurrence of HCC. They also confirmed that patients with HCC showed significantly higher PTX3 concentrations compared with patients with mild or severe fibrosis. Interestingly, the study by Song et al. [75] conducted using tissues also showed increased PTX3 expression in tumor tissues compared with normal tissues. This expression correlated with an increased serum AFP concentration, tumor size, cirrhosis, stage of cancer advancement, presence of metastases, PVTT (portal vein tumor thrombosis), and MVI (microvascular invasion). These data indicated that PTX3 could accelerate HCC progression through activating epithelial–mesenchymal transition and serve as a potential predictive factor and therapeutic target for HCC. Similarly, in the case of research conducted by Cabiati et al. [76] using tissues, PTX3 was upregulated in HCC tissues. The authors noted that this parameter could be used to assess the advancement of the disease and its use as a staging marker in HCV-related HCC cases should be considered. In contrast to the reported results, we found one study that indicated the lack of any usefulness of Pentraxin 3 in patients with HCC [77]. However, this work was performed using the serum of only 31 patients. Interestingly, in the discussion section, the authors suggested that the liver could eliminate PTX3 from the blood, which seems to be an interesting assumption, but requires confirmation [77].

The usefulness and role of Pentraxin 3 in esophageal cancer were described by Fan et al. [78] and Ma et al. [79]. The authors showed that the overexpression of PTX3 in esophageal cancer cell lines reduced cell proliferation, colony formation, cell cycle, ability to migrate, as well as sensitivity to radio- and chemotherapy. Moreover, Fan et al. [78] confirmed their assumptions by repeating the study on the PTX3 gene knockout model, where they observed directly opposite relationships. Moreover, PTX3 gene knockout also decreased cell glycolysis and increased oxidative phosphorylation, which is consistent with other findings that PTX3 affects the tumorigenic ability of cells and their sensitivity to anticancer treatment. In the case of the work by Ma et al. [79], it was also proven using a xenograft study that increased the expression of PTX3 in esophageal cancer cell lines inhibited tumor growth in vivo. The remaining works describing the involvement of PTX3 in esophageal cancer concerned the description of the changes in the concentration of this parameter as a prognostic factor after adding herbs (Jie Du Tong Ye San) [80] and freeze-dried black raspberries or their polyphenolic anthocyanins [81] to the diet of the examined rats. Interestingly, both the traditional Chinese herbal formula and black raspberries are considered to have anti-cancer properties. The above-mentioned studies showed that PTX3 can serve as an anti-inflammatory marker due to its highly downregulated expression in esophageal cancer. The administration of the mentioned substances resulted in an increase in PTX3 concentration compared with the concentrations obtained in the plasma of rats on a control diet, which confirms the above-mentioned assumptions [80,81].

In the work of Cui et al. [82], the authors demonstrated that PTX3 upregulation inhibited the stemness of gastric cancer (GC) with the use of GC cell lines. They also revealed a new function of PTX3 in promoting the alternative activation of TAMs (tumor-associated macrophages) in which PTX3 suppressed the differentiation of M2 macrophages by decreasing the expression of IL-4 and IL-10 via the JNK1/2 pathway. These findings suggested the biological role of PTX3 in the progression of GC, indicating an investigational basis for its usage as a tumor biomarker and potential target for the treatment of GC. Interestingly, Yeni et al. [83] revealed that the serum concentrations of PTX3 (similar to VEGF and IL-8) were statistically lower in the group of patients with stomach cancer than in healthy participants. The authors explained their reports by the assumption that they were connected to a deficiency or suppression of PTX3, which behaves as an oncosuppressor; for this reason, it can play a role in the development of gastric adenocarcinoma. In addition, correlation tests, irrespective of groups, showed that serum PTX3, IL-8, and VEGF affected one another in gastric adenocarcinomas. On the contrary, in the work by Choi et al. [84], the authors reported that the concentration of Pentraxin 3 in gastric cancer was increased and the suppression of PTX3 in GC cell lines caused the inhibition of chemotactic migration of macrophages. These findings provide a better understanding of the mechanisms by which PTX3 exerts its effects on the progression of gastric cancer-related inflammation. The same research team, in their second work, revealed a functional interaction between BDNF/TrkB and PTX3 in advanced gastric cancer and provided evidence that these proteins may enhance the interaction between bone metastatic gastric cancer cells and osteoblasts leading to osteolysis, and that their expression might allow the prediction of metastatic potential and improve the prognosis of patients with advanced gastric cancers. Due to significant discrepancies between published works, it is difficult to clearly confirm the direction of action of PTX3 in gastric cancer [85]. 

In the paper by Goulart et al. [86] concerning pancreatic cancer, the authors revealed that high serum PTX3 concentrations (>4.34 ng/mL) had higher diagnostic utility (sensitivity, specificity, positive predictive value, and likelihood ratio) than routine tumor markers, such as CA 19-9 and CEA. The in vitro and ex vivo analyses of Pentaxin 3 performed by the authors suggested that it originated from stromal cells. PTX3 organizes hyaluronan in conjunction with tumor necrosis factor-stimulated gene 6 (TSG-6) and facilitates stellate and cancer cell invasion. Therefore, Pentaxin 3 seems to be an important parameter useful in the diagnosis, detection, and treatment of pancreatic cancer. Similar reports were presented in the work of Kamal et al. [87], where the authors confirmed high PTX3 concentrations in patients with pancreatic cancer compared with healthy patients. The authors emphasized that its high diagnostic usefulness can be quantified in the systemic circulation and may be useful in selecting patients for further diagnostic imaging. These results were confirmed by additional tests that showed higher expression of this protein in tumor tissue and in surgical tissues of oncological patients. Moreover, PTX3 is already differentially expressed in patients with early stage pancreatic ductal adenocarcinoma and in patients with CA19-9 levels below the standard threshold. Other papers also confirmed that PTX3 expression is upregulated in activated pancreatic stellate cells and can be used as a potential diagnostic biomarker for pancreatic cancer [88]. Similar results were presented by Kondo et al. [89], who revealed that PTX3 levels were elevated in pancreatic carcinoma cells in culture solution, which was considered an indication of direct PTX3 secretion by these cells. The authors also confirmed a strong correlation between pentraxin 3 expression and the prognosis of pancreatic carcinoma. In addition, in the work by Rosendahl et al. [90], the authors found that the inflammatory response mediator Pentraxin 3 (PTX3) is almost eight-fold upregulated in tumor-derived versus non-tumor human pancreatic stellate cells. In contrast with these reports, in the paper by McCarthy et al. [91] performed with the use of cell lines, the loss of the RNA-binding protein human antigen R resulted in the downregulated secretion of protein PTX3 by 92.3%. This may confirm that Pentraxin 3 has a protective and anti-cancer function. Interestingly, we also found a paper concerning Pentraxin 3 as an adipose tissue-related serum marker for pancreatic cancer cachexia. The authors proved that high serum PTX3 was an independent risk factor for visceral fat loss, decreased skeletal muscle mass index, and poor prognosis for pancreatic cancer patients [92].

We did not find any studies describing the involvement of PTX3 in the course of gallbladder, anus, and small intestine cancers. A summary of the changes in serum PTX3 concentrations, tissue expression or concentrations in tissue homogenates, and gene expression using cell lines is presented in Table 1.

Taking into account the previously described publications on the role of PTX3 in the course of gastrointestinal cancers, conclusions can be drawn about the significant role of this parameter in their course. Due to the fact that the mechanism of action of Pentraxin 3 can be described as pro- and anti-cancer, its specific role in the course of individual cancers is difficult to determine. However, in most cases, the expression/concentration of this parameter is increased in the presence of cancer lesions, which correlates with a poor survival rate and the stage of advancement of cancer lesions, suggesting the secretion of this protein by cancer cells. A significant correlation of PTX3 with complement components was also observed, which may suggest a correlation with the activation of the complement system. The mechanism of this action has already been presented (Figure 1—intensification of activation of the complement pathway and promotion of inflammatory processes), which, together with the second function of PTX3, i.e., intensification of angiogenesis processes, leads to the rapid development of cancer lesions.

## 4. Literature Search and Data Extraction

**Figure 2:**
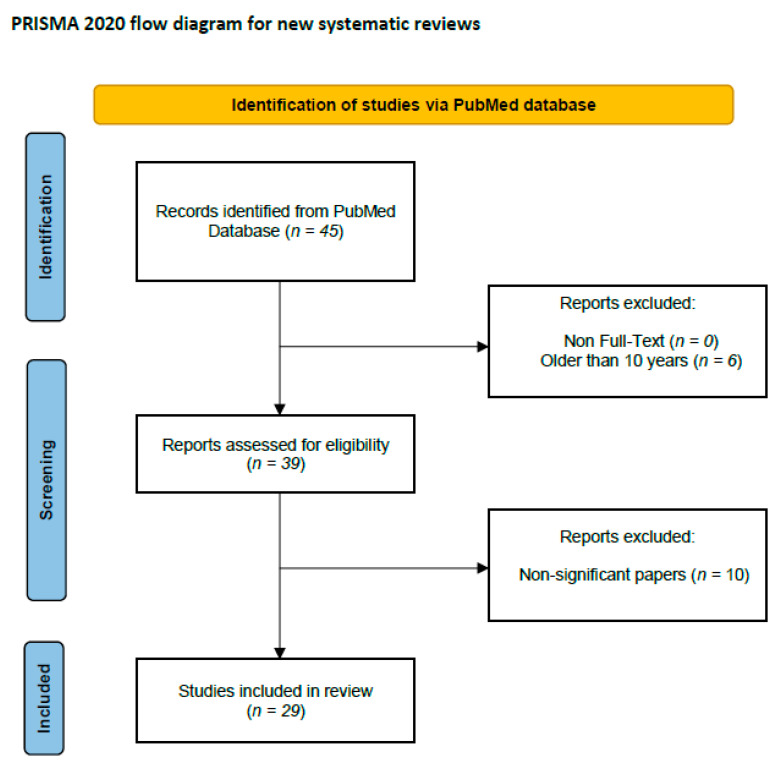
Schematic illustration of articles included in the review.

We performed a comprehensive literature search using the MEDLINE/PubMed electronic database on 21 October 2023. Table 2 and Figure 2 were added to explain the search strategy for this systematic review. This review was not registered in PROSPERO at inception. For the first data search, we found 45 papers. We also applied two additional filters: Full-Text in Text Availability and 10 years for Publication Date. In the case of the use of these data search exclusions, we found 39 papers. In the next step, we excluded all papers that were non-significant (papers not concerning PTX3, GI cancers, duplicated, or retracted). Finally, 29 publications were included in the study. All steps were included in the PRISMA 2020 Flow Diagram (Figure 2) [93]. 

## 5. Conclusions

Cancers of the digestive tract, which include cancer of the esophagus, stomach, liver and gallbladder, pancreas, small intestine, colon, and rectum, constitute a very important problem all over the world due to the emerging number of new cases. Due to the increasing need to search for new biomarkers and therapeutic targets necessary in the detection and treatment of cancerous lesions, we attempted to determine the role of Pentraxin 3 in their course. These proteins belong to the family of fast-reacting proteins, such as C-reactive protein, whose usefulness in cancer has been widely proven. Pentraxin 3 is involved in angiogenesis and inflammatory processes. In the course of gastrointestinal cancers, there are statistically significant changes in the concentration of this parameter. Moreover, the increase in its concentration is related to the advancement of cancer and processes leading to the development of cancerous lesions (e.g., in the case of liver diseases, an increase in concentration is observed in cirrhosis, which progresses further due to HCC initiation). In the case of studies concerning tissue material, both increased and decreased tissue expression of the tested parameter was observed, depending on the type of cancer (detailed information can be found in Table 1). In the case of cell lines, both human and animal cell lines, a significant increase in PTX3 expression was observed, which confirmed the changes in the protein concentrations. To conclude, it can be assumed that PTX3, both at the level of gene expression and protein concentrations, is highly useful in detecting cancer lesions (also in the case of low-advanced changes) in the course of gastrointestinal cancers, and its use as a biomarker and therapeutic target may be an important element in the development of modern medicine.

## Figures and Tables

**Table 1:** Summary of Pentraxin 3 changes in gastrointestinal cancers.

Material/Cancer Type	Serum/Plasma	Tissue	Cell Line
Colorectal cancer	↑	n/s	↑
Liver cancer	↑	↑	-
Esophageal cancer	↓	↓	↑
Pancreatic cancer	↑	↑	↑
Gastric cancer	↓/↑	-	↑

Abbreviations: n/s—non-significant differences; ↑—raised in cancer; ↓—lowered in cancer.

**Table 2:** Literature search strategy.

Entry	Number of Available Publications	Number of Publications from Last 10 Years with Full Text	Number of Irrelevant Publications	Number of Publications Taken into Analysis
(colorectal/colon cancer) AND (pentraxin 3)	10	10	1 (irrelevant)	9
(liver cancer) AND (pentraxin 3)	17	13	7 (irrelevant)	6
(esophageal cancer) AND (pentraxin 3)	5	4	0	4
(pancreatic cancer) AND (pentraxin 3)	8	7	1 (retracted)	6
(gastric cancer) AND (pentraxin 3)	5	5	1 (retracted)	4
(gallbladder cancer) AND (pentraxin 3)	0	0	0	0
(anus cancer) AND (pentraxin 3)	0	0	0	0
(small intestine cancer) AND (pentraxin 3)	0	0	0	0
